# Lipoprotein dynamics in neuromyelitis optica spectrum disorder

**DOI:** 10.1016/j.jlr.2025.100864

**Published:** 2025-07-21

**Authors:** Tsai-Wei Liu, Mei-Ling Cheng, Chiung-Mei Chen, Long-Sun Ro, Kuo-Hsuan Chang

**Affiliations:** 1 Center of Neuroimmunology and Rare Diseases, Section of Neuromuscular Disorders, Department of Neurology, Chang Gung Memorial Hospital Linkou Medical Center, Taoyuan City, Taiwan; 2 Department of Biomedical Sciences, Chang Gung University, Taoyuan City, Taiwan; 3 Metabolomics Core Laboratory, Healthy Aging Research Center, Chang Gung University, Taoyuan City, Taiwan; 4 School of Medicine, Chang Gung University, Taoyuan City, Taiwan

**Keywords:** neuromyelitis optica spectrum disorder, multiple sclerosis, lipoprotein, autoimmune disorder

## Abstract

Neuromyelitis optica spectrum disorder (NMOSD) is a neuroinflammatory disease caused by aquaporin-4 IgG antibodies, which damage astrocytes and trigger inflammation. Although altered lipid profiles have been observed in various neuroinflammatory diseases, the role of dyslipidemia in NMOSD disease activity remains poorly understood. In this study, we analyzed plasma lipoprotein profiles in 40 patients with NMOSD during relapses, 35 patients with multiple sclerosis (MS) during relapses, and 41 age- and sex-matched healthy controls (HCs). Among 112 lipoprotein components, 39 showed significant alterations in NMOSD patients compared with both MS patients and HCs. These components exhibited consistently lower levels during relapses. Receiver operating characteristic analysis identified total apolipoprotein-A2 (Apo-A2; area under the curve [AUC] = 0.808), HDL-3-Apo-A2 (AUC = 0.806), HDL-Apo-A2 (AUC = 0.798), VLDL-2-phospholipids (PLs; AUC = 0.774), VLDL-3-PLs (AUC = 0.769), and VLDL-3-triglycerides (AUC = 0.770) as robust biomarkers for distinguishing NMOSD from HCs, whereas VLDL-3-PLs (AUC = 0.791) and HDL-3-Apo-A2 (AUC = 0.752) effectively differentiated NMOSD from MS. Importantly, HDL-4-Apo-A2 levels negatively correlated with Expanded Disability Status Scale scores (*r* = −0.321, *P* = 0.043) and spinal cord lesion length (*r* = −0.391, *P* = 0.013) in NMOSD patients. Among 22 NMOSD patients evaluated longitudinally, 36 of the 39 dysregulated lipoprotein components return to normal levels during remission. This study represents the first comprehensive lipidomic analysis in NMOSD, revealing distinct dyslipidemia patterns associated with disease activity and highlighting the potential of lipoprotein profiling as a noninvasive prognostic biomarker.

Neuromyelitis optica spectrum disorder (NMOSD) is a chronic neuroinflammatory disease characterized by longitudinally extensive transverse myelitis and optic neuritis (1). Patients with NMOSD experience a relapsing-remitting course, resulting in multiple inflammatory lesions in the central nervous system (CNS) and substantial morbidity. Initially considered a variant of multiple sclerosis (MS), NMOSD was reclassified as a distinct entity following the identification of autoantibodies targeting aquaporin-4 (AQP4), a water channel protein on astrocytes (2, 3). These AQP4-IgG autoantibodies drive complement-mediated inflammation and astrocyte necrosis within the CNS, in contrast to MS, where T-cell-mediated immune responses predominantly target myelin and oligodendrocytes (1, 4, 5).

Lipoproteins are macromolecular complexes composed of triglycerides (TGs), cholesterol (CH), phospholipids (PLs), and apolipoproteins, responsible for transporting lipids through blood or other extracellular fluids to cells via receptor-mediated endocytosis (6). Based on their density, size, lipid composition, and apolipoprotein content, lipoproteins are classified into high density lipoproteins (HDLs), low density lipoproteins (LDLs), intermediate density lipoproteins (IDLs), and very low density lipoproteins (VLDLs) (7). Advances in analytical techniques have enabled detailed characterization of lipoprotein subfractions (e.g., HDL-1, HDL-2, HDL-3, HDL-4, LDL-1, LDL-2, LDL-3, LDL-4, LDL-5, LDL-6, VLDL-1, VLDL-2, VLDL-3, VLDL-4, VLDL-5) and quantification of lipid and protein components, including total cholesterol (TC), free cholesterol (FC), CH, PL, TG, apolipoprotein (Apo-A1, Apo-A2, Apo-B, and Apo-B100), and particle number (PN). These advancements highlight the diagnostic and prognostic potential of specific lipoprotein profiles. HDL particles undergo dynamic remodeling during reverse CH transport. Initially, small HDL-3 particles form by acquiring PLs from peripheral tissues and are then converted into larger HDL-2 particles, which are associated with cardioprotective effects (8). On the other hand, the metabolic transformation of VLDL, IDL, and LDL follows distinct pathways that produce various IDL and LDL subfractions from TG-rich lipoprotein precursors (9). Large VLDL particles undergo stepwise lipolysis, yielding small IDL and subsequently small, dense LDL particles (10). These LDL particles have atherogenic potential due to their low affinity for LDL receptor and prolonged circulation (11). Their enhanced capacity to penetrate arterial walls, bind to proteoglycans, and undergo oxidative modification promotes macrophage uptake, CH accumulation, and atherosclerosis (11). By contrast, HDL particles facilitate reverse CH transport from peripheral tissues, exhibit vasodilatory, anti-inflammatory, and endothelial protective properties, which help prevent the development of atherosclerosis (12).

Emerging evidence demonstrates that altered lipid metabolism plays a role in MS pathogenesis (13, 14, 15, 16, 17). Elevated LDL levels are associated with the development of new T2 lesions on brain MRI (13). Similarly, increased Apo-B levels correspond to a higher burden of new and enlarging T2 lesions, emphasizing the contribution of proatherogenic lipoproteins to MS disease activity (15). Higher baseline LDL levels are associated with greater disability progression (14). Increases in HDL-CH and Apo-A1 are related to slower rates of gray matter and cortical volume loss (17). Pathological studies reveal colocalization of LDL within foamy macrophages in actively demyelinating MS plaques, supporting the involvement of lipid dysregulation in lesion formation and disease progression (18). These findings highlight the complex interplay between lipid metabolism, neuroinflammation, and MS pathophysiology.

Given the shared relapsing-remitting course and neuroinflammatory nature of NMOSD and MS, lipid metabolism disturbances may similarly contribute to NMOSD disease activity. However, comprehensive lipoprotein profiling in NMOSD has not been investigated. This study aims to characterize plasma lipoprotein profiles in NMOSD patients during relapse and remission, compare them with those of MS patients and healthy controls (HCs), and evaluate their potential as biomarkers of disease activity and differential diagnosis.

## Materials and Methods

### Ethics approval and consent to participate

This study was approved by the Institutional Review Board of Chang Gung Memorial Hospital (approval numbers: 201302260A3D001, 201701423B and 202201755B0). Venous blood samples were collected from participants with NMOSD, MS, and HCs. Informed consent was obtained from all participants following a detailed explanation of the study objectives.

### Patient recruitment

This study was conducted at Chang Gung Memorial Hospital-Linkou Medical Center in Taiwan from January 1, 2017 to December 31, 2022. Patients with NMOSD or MS were recruited and diagnosed according to the international consensus diagnostic criteria for NMOSD (19) and the McDonald criteria for MS (20). Both AQP4-IgG-positive and -negative patients were eligible for inclusion. Exclusion criteria included systemic infection, chronic renal failure, cardiac or liver dysfunction, malignancies, or autoimmune diseases other than NMOSD or MS. Age- and sex-matched HCs were randomly recruited from neurology outpatient clinics. None of the patients with NMOSD, MS, or HCs received statins or other lipid-lowering therapies at the time of blood sampling.

### Sample collection and plasma preparation

Venous blood samples were collected via venipuncture from all participants. For NMOSD and MS patients, “relapse” samples were obtained within 2 weeks of symptom onset during an acute relapse, before administration of corticosteroids, intravenous immunoglobulins, or plasmapheresis. Fasting blood samples, collected after at least 8 h of fasting, were obtained from 36 NMOSD patients, 30 MS patients, and all HCs. Due to urgent treatment needs during relapses, samples from four NMOSD patients and five MS patients were collected without fasting. “Remission” samples were collected in a fasting state, at least 3 months after symptom onset and no later than 1 month before the next relapse. Blood samples were maintained at room temperature for 30 min, then centrifuged at 1,000–2,000 *g* for 10 min. Plasma was separated from the supernatant, aliquoted, frozen at −80°C, and stored for subsequent analysis. Neurological disability was evaluated at the time of venipuncture for both relapse and remission samples using the Kurtzke Expanded Disability Status Scale (EDSS) (21).

### Nuclear magnetic resonance analysis (^1^H-NMR spectroscopic measurements)

Plasma samples (100 μl) were diluted 1:1 with 75 mM sodium phosphate (pH 7.4), and 200 μl of the mixture was transferred into a 3-mm Bruker SampleJet NMR tube (Bruker Biospin GmbH, Rheinstetten, Germany). NMR analysis was performed using Bruker Avance III HD 600 MHz spectrometers equipped with a TXI probe. Sample temperature was maintained at 6°C via the Bruker SampleJet system. For each sample, a solvent-presaturation 1D 1H experiment (64 scans, 98,304 data points, spectral width 18,028.85 Hz) was followed by a 1D 1H Carr-Purcell-Meiboom-Gill spin-echo experiment (64 scans, 73,728 data points, spectral width 12,019.23 Hz). Data were acquired and processed using Bruker Topspin 3.6.2 and ICON NMR, which automatically performed phasing, baseline correction, and calibration (TSP to 0 ppm). Lipoprotein subfraction analysis, encompassing 112 subfractions, was conducted using Bruker IVDr Lipoprotein Subclass Analysis (B.I.-LISA) method. Peak intensities of -CH2 (δ = 1.25 ppm) and -CH3 (δ = 0.8 ppm) in the 1D spectrum were quantified, normalized with the Bruker QuantRef manager in Topspin, and analyzed using a partial least squares regression model (22). This provided data on Apo-A1, Apo-A2, Apo-B, Apo-B100, CH, FC, PL, PN, and TG in different classes of density: VLDL (density 0.950–1.006 kg/l), LDL (density 1.019–1.063 kg/l), IDL (density 1.006–1.019 kg/l), and HDL (density 1.063–1.210 kg/L). VLDL, LDL, and HDL were further subdivided into five, six, and four density subfractions, respectively (23). All instrumentation and software were provided by Bruker Biospin GmbH (Rheinstetten, Germany).

### Statistical analysis

Continuous variables were presented as mean ± standard deviation and analyzed by Student’s *t-*test or ANOVA, with adjustments for multiple comparisons using the false discovery rate method or Bonferroni post hoc correction, as appropriate. Categorical variables were reported as counts and percentages and analyzed using the Chi-square test. Pearson’s correlation was used to evaluate the associations between molecular levels and clinical parameters. Receiver operating characteristic (ROC) curve analysis evaluated the ability of individual components to differentiate MS and NMOSD patients from HCs. Selected molecules were further analyzed using a support vector machine algorithm, with model performance evaluated through ROC curves generated by Monte-Carlo crossvalidation with balanced subsampling. One hundred crossvalidations were performed, and averaged results generated the final plot. All statistical analyses were conducted using R software, version 4.0.3 with rstatix and MetaboAnalyst 6.0 packages (McGill University, Montreal, Canada).

## Results

The demographic characteristics of the study groups are summarized in Table 1. We recruited 40 patients with NMOSD (31 women, 9 men), 35 patients with MS (23 women, 12 men), and 41 sex- and age-matched HCs (23 women, 18 men). Among NMOSD patients, 37 were AQP4-IgG positive and 3 were AQP4-IgG negative. MS patients (mean age: 36.77 ± 14.39 years) were significantly younger than NMOSD patients (46.45 ± 14.99 years, *P* = 0.009) and HCs (46.56 ± 14.07 years, *P* < 0.001). NMOSD patients exhibited significantly higher EDSS scores (4.59 ± 2.22, *P* < 0.001) compared with MS patients (3.51 ± 2.25). NMOSD relapses were predominantly characterized by longitudinally extensive spinal cord lesions (87.50%, n = 35) and optic neuritis (67.50%, n = 27).

**Table 1:** Clinical characteristics of patients with NMOSD, MS, and HCs

Parameter	NMOSD (N = 40)	MS (n = 35)	HC (n = 41)
Sex (female/male)	31/9	23/12	23/18
Age (years)	46.45 ± 14.99	36.77 ± 14.39^a^^b^	46.56 ± 14.07
Sugar AC (B) (mg/dl)	105.61 ± 24.11	97.10 ± 22.06	98.55 ± 22.06
TC (B) (mg/dl)	161.43 ± 30.77	171.66 ± 26.18	164.32 ± 26.30
Total TG (B) (mg/dl)	91.95 ± 39.73	142.15 ± 78.23	120.76 ± 101.81
EDSS	4.59 ± 2.22^c^	3.51 ± 2.25	
LESCL (%)	35 (87.50)	1 (2.9)	
ON (%)	27 (67.50)	5 (14.29)	
Infratentorial (%)	15 (37.5)	12 (34.3)	
AQP4-IgG (%)	37 (92.5)	0 (0)	
CSF			
WBC (cells/μl)	6.38 ± 13.33	1.40 ± 2.42^c^	
Oligoclonal bands	0 (0)	11 (31.43)	
IgG index	0.68 ± 0.98	0.90 ± 0.64	
Medications			
Interferon-β1a		5 (14.29)	
Dimethyl fumarate		17 (48.57)	
Fingolimod		11 (31.43)	
Cladribine		2 (5.71)	
Prednisolone	20 (50.00)		
Azathioprine	15 (37.50)		
Mycophenolate	5 (12.50)		

LESCL, longitudinally extensive spinal cord lesion; ON, optic neuritis.

a Statistically significant in comparison with NMOSD (MS vs. NMOSD). *P* < 0.05, Bonferroni post hoc adjustment.

b Statistically significant in comparison with HC (MS vs. HC). *P* < 0.05, Bonferroni post hoc adjustment.

c Statistically significant in comparison with NMOSD (MS vs. NMOSD). *P* < 0.05, two-tailed Student’s *t-*test.

**Fig. 1:**
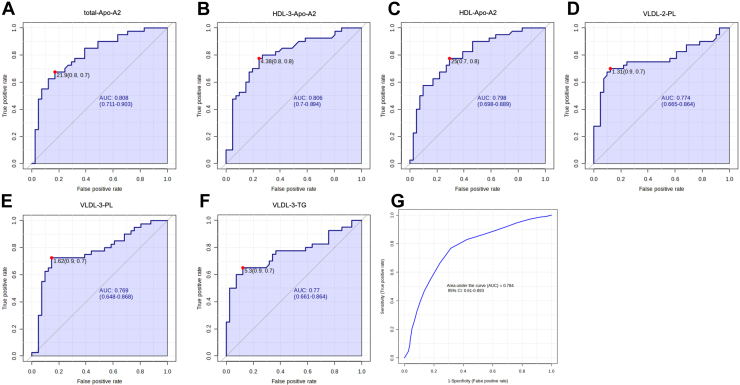
Differentiation potential of lipoprotein components between NMOSD and HCs. A: Total-Apo-A2, (B) HDL-3-Apo-A2, (C) HDL-Apo-A2, (D) VLDL-2-PL, (E) VLDL-3-PL, and (F) VLDL-3-TG exhibit strong discriminatory ability in distinguishing NMOSD from HCs. The analysis highlights the AUC for the six most discriminative lipoprotein components (AUC >0.75). G: ROC curves of the six components are shown based on crossvalidation (CV) performance.

**Fig. 2:**
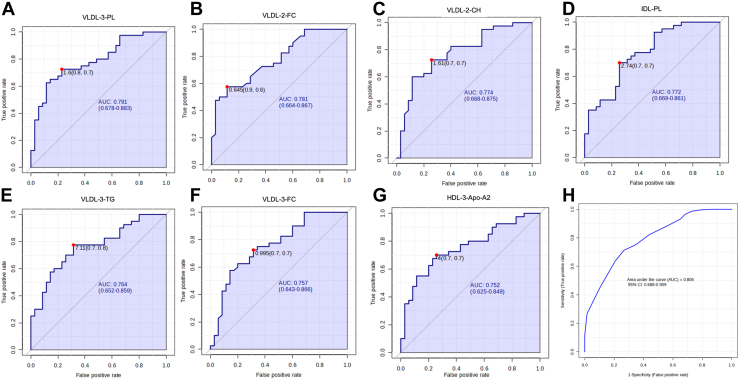
Differentiation potential of lipoprotein components between NMOSD and multiple sclerosis (MS). A: VLDL-3-PL, (B) VLDL-2-FC, (C) VLDL-2-TC, (D) IDL-PL, (E) VLDL-3-TG, (F) VLDL-3-FC, (G) HDL-3-Apo-A2 effectively differentiate NMOSD from MS. The analysis highlights the AUC for the seven highest lipoprotein components (AUC >0.75), (H) ROC curves of these seven lipoprotein components are displayed based on crossvalidation (CV) performance.

Plasma levels of 112 lipoprotein components were quantified, with 39 showing significant differences among NMOSD, MS, and HCs (Table 2 and supplemental Table S1). These alterations were observed in PN, Apo-A1, Apo-A2, and Apo-B100 across total plasma (total-Apo-A1/Apo-A2/Apo-B100/PN), HDL subfractions (HDL-TG/Apo-A2, HDL-2-Apo-A2/TG, HDL-3-Apo-A2/TG, and HDL-4-Apo-A2), IDL subfractions (IDL-Apo-B100/CH/FC/PL/PN), LDL subfractions (LDL-5-TG, LDL-6-CH/FC/PL/PN/Apo-B100), and VLDL subfractions (VLDL-CH/FC/PL/TG, VLDL-1-FC, and VLDL-2/3/4-CH/FC/PL/TG). NMOSD patients exhibited significantly lower levels of these lipoprotein components compared with both MS patients and HCs. The most pronounced differences between NMOSD and MS were observed in total-Apo-A2, HDL-Apo-A2, and HDL-3-Apo-A2. ROC analysis identified several components with strong discriminatory power between NMOSD and HCs, such as total-Apo-A2 (area under the curve [AUC] = 0.808), HDL-3-Apo-A2 (AUC = 0.806), HDL-Apo-A2 (AUC = 0.798), VLDL-2-PL (AUC = 0.774), VLDL-3-PL (AUC = 0.769), and VLDL-3-TG (AUC = 0.77; Fig. 1). Markers for differentiating NMOSD from MS were also identified, including VLDL-3-PL (AUC = 0.791), VLDL-2-FC (AUC = 0.781), VLDL-2-CH (AUC = 0.774), IDL-PL (AUC = 0.772), VLDL-3-FC (AUC = 0.757), and HDL-3-Apo-A2 (AUC = 0.752; Fig. 2).

**Table 2:** Significantly differentiated lipoprotein plasma levels in patients with NMOSD compared with MS and HCs

Metabolite (mg/dl, mean ± SD)	HC (n = 41)	NMOSD (n = 40)	MS (n = 35)	*P* value^a^ (3 groups)	*P* value^b^ (NMOSD vs. HC)	*P* value^c^ (NMOSD vs. MS)
Total-Apo-A1	136.76 ± 26.58	117.57 ± 22.72	129.84 ± 20.49	0.002	0.001	0.017
Total-Apo-A2	27.44 ± 2.92	20.06 ± 5.67	24.26 ± 4.85	<0.001	<0.001	0.001
Total-Apo-B100	74.26 ± 18.36	65.01 ± 14.62	73.24 ± 16.43	0.027	0.014	0.025
Total-PN	1,350.23 ± 333. 88	1,182.13 ± 265.81	1,331.78 ± 298.83	0.027	<0.001	0.001
HDL-2-Apo-A2	2.90 ± 1.49	1.94 ± 0.96	2.96 ± 1.32	0.001	0.003	<0.001
HDL-2-TG	1.84 ± 0.93	1.43 ± 0.74	1.90 ± 0.78	0.027	0.272	0.009
HDL-3-TG	2.10 ± 0.94	1.60 ± 0.69	2.09 ± 0.81	0.010	0.016	0.006
HDL-3-Apo-A2	5.55 ± 1.94	3.60 ± 1.36	4.89 ± 1.53	<0.001	<0.001	<0.001
HDL-4-Apo-A2	5.55 ± 1.94	3.60 ± 1.36	4.89 ± 1.53	<0.001	<0.001	0.337
HDL-Apo-A2	28.46 ± 6.69	21.57 ± 5.24	25.66 ± 4.81	<0.001	<0.001	0.001
HDL-TG	10.07 ± 4.53	8.67 ± 3.69	11.04 ± 4.11	0.025	0.026	0.010
IDL-Apo-B100	3.87 ± 2.74	2.35 ± 1.46	4.16 ± 2.79	0.002	0.002	0.001
IDL-CH	9.95 ± 8.58	5.23 ± 3.74	10.47 ± 7.88	0.002	0.001	<0.001
IDL-FC	2.84 ± 2.46	1.49 ± 1.02	3.02 ± 2.18	0.001	0.002	<0.001
IDL-PL	70.36 ± 49.81	42.71 ± 26.62	75.69 ± 50.80	<0.001	<0.001	<0.001
IDL-PN	70.36 ± 49.81	42.71 ± 26.63	75.69 ± 50.80	0.002	0.001	0.001
LDL-5-TG	1.16 ± 1.95	1.27 ± 0.89	1.87 ± 0.82	0.004	0.001	0.003
LDL-6-Apo-B100	16.84 ± 4.54	13.47 ± 4.54	18.24 ± 7.40	0.013	0.033	0.001
LDL-6-CH	19.91 ± 10.18	15.26 ± 5.78	20.40 ± 7.81	0.011	0.014	0.002
LDL-6-FC	4.84 ± 2.12	3.72 ± 1.48	4.57 ± 1.66	0.015	0.007	0.011
LDL-6-PL	11.24 ± 4.96	9.06 ± 2.88	11.63 ± 3.78	0.011	0.018	0.001
LDL-6-PN	306.11 ± 158.71	244.92 ± 82.48	331.58 ± 134.51	0.013	0.033	0.001
VLDL-1-FC	2.41 ± 3.33	1.16 ± 1.10	2.78 ± 2.44	0.013	0.027	<0.001
VLDL-2-CH	2.54 ± 2.27	1.45 ± 1.15	2.92 ± 1.72	<0.001	0.006	<0.001
VLDL-2-FC	1.35 ± 1.32	0.78 ± 0.61	1.65 ± 1.06	0.002	0.018	<0.001
VLDL-2-PL	2.57 ± 1.97	1.39 ± 1.13	2.48 ± 1.51	0.001	<0.001	0.001
VLDL-2-TG	9.72 ± 7.95	5.93 ± 4.58	9.85 ± 6.33	0.011	<0.001	0.003
VLDL-3-CH	2.97 ± 2.44	1.69 ± 1.29	3.28 ± 2.24	0.002	0.005	<0.001
VLDL-3-FC	1.52 ± 1.48	0.85 ± 0.67	1.80 ± 1.29	0.003	0.016	<0.001
VLDL-3-PL	3.22 ± 2.14	1.72 ± 1.25	3.35 ± 1.92	<0.001	<0.001	<0.001
VLDL-3-TG	9.57 ± 5.95	5.51 ± 4.13	10.14 ± 5.62	<0.001	<0.001	<0.001
VLDL-4-CH	4.42 ± 2.52	2.87 ± 1.73	4.76 ± 2.63	0.001	0.006	<0.001
VLDL-4-FC	1.83 ± 1.31	1.10 ± 0.74	2.11 ± 1.43	0.001	0.009	<0.001
VLDL-4-PL	4.15 ± 1.81	2.80 ± 1.47	4.34 ± 2.07	<0.001	0.001	<0.001
VLDL-4-TG	8.72 ± 3.64	6.12 ± 3.10	9.21 ± 4.44	0.001	0.001	0.001
VLDL-CH	20.11 ± 16.47	13.13 ± 7.07	22.96 ± 12.81	0.003	0.016	0.003
VLDL-FC	9.52 ± 6.72	7.19 ± 3.02	11.05 ± 6.72	0.010	0.027	<0.001
VLDL-PL	19.76 ± 13.77	13.90 ± 6.12	21.82 ± 11.79	0.006	0.023	<0.001
VLDL-TG	75.02 ± 28.65	47.87 ± 28.65	85.96 ± 57.08	0.011	0.030	<0.001

a *P* value by ANOVA with false discovery rate correction.

b *P* value by Bonferroni post hoc adjustment.

c *P* value by Bonferroni post hoc adjustment.

**Fig. 3:**
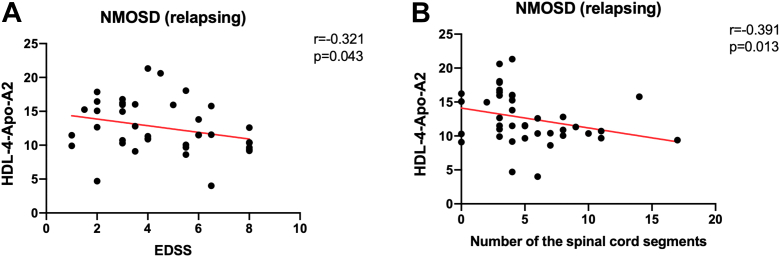
Correlation between HDL-4-Apo-A2 levels and disease severity in NMOSD. A: Correlation between plasma levels of HDL-4-Apo-A2 and the scores on the Kurtzke EDSS. B: Correlation between plasma levels of HDL-4-Apo-A2 and the spinal cord lesion length in patients with NMOSD.

To assess whether these lipoproteins could serve as markers of disease progression, we analyzed their correlation with EDSS scores and spinal cord lesion length. Plasma HDL-4-Apo-A2 levels demonstrated a significant negative correlation with EDSS (*r* = −0.321, *P* = 0.043; Fig. 3A) and spinal cord lesion length (*r* = −0.391, *P* = 0.013; Fig. 3B).

**Fig. 4:**
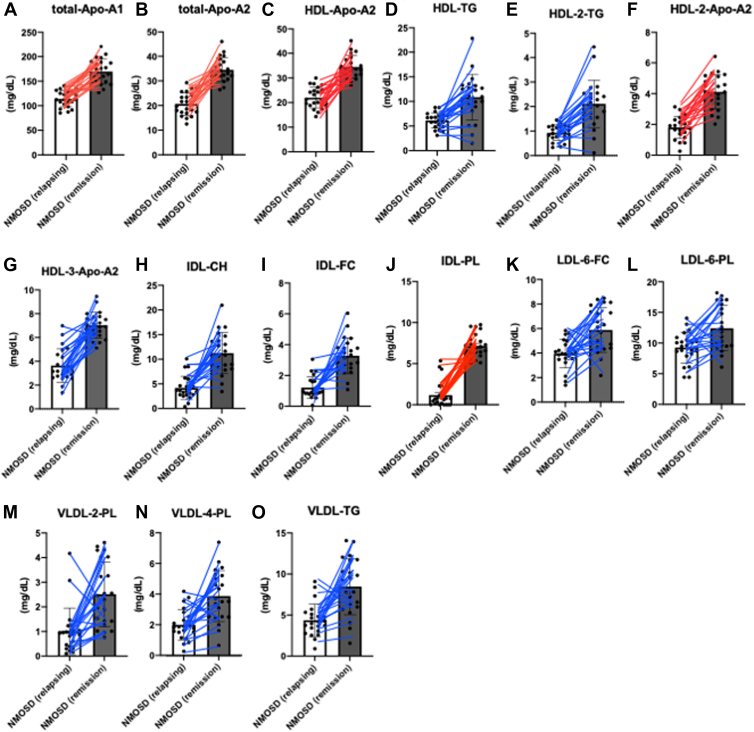
Lipoprotein component normalization during remission in NMOSD (A, B) total apolipoproteins (total-Apo-A1, total-Apo-A2); (C–G) HDL components (HDL-TG, HDL-2-TG, HDL-2-Apo-A2, and HDL-3-Apo-A2); (H–J) IDL components (IDL-CH, IDL-FC, and IDL-PL); (K, L) LDL-6 components (LDL-6-FC, LDL-6-PL); (M–O) VLDL components (VLDL-2-PL, VLDL-4-PL, and VLDL-TG). The components were selected and presented here based on significant changes between the relapsing and remission phases observed in over 85% of patients. Red line: Lipoprotein levels that normalized to ranges comparable to those of HCs during remission in the majority of patients. Blue line: Lipoprotein levels that remained decreased or did not fully normalize in a subset of patients during remission.

In 22 NMOSD patients with both relapse and remission samples, 36 of 39 lipoprotein components that were significantly decreased during relapses normalized to levels comparable to HCs during remission. These components included total-Apo-A1/Apo-A2/Apo-B100/PN, HDL-Apo-A2/TG, HDL-2-Apo-A2/TG, HDL-3-Apo-A2/TG, HDL-4-Apo-A2, IDL-Apo-B100/CH/FC/PL/PN, LDL-5-TG, LDL-6-CH/FC/PL/PN/Apo-B100), and VLDL-CH/TG, VLDL-1-FC, and VLDL-2/3/4-CH/FC/PL/TG. The most significant changes between relapse and remission were observed in IDL-PL, HDL-3-Apo-A2, and total-Apo-A2 (Table 3 and Fig. 4).

**Table 3:** Comparison of lipoprotein plasma levels between patients with NMOSD during relapse and remission

Mean ± SD (mg/dl, mean ± SD)	NMOSD (relapsing) (n = 22)	NMOSD (remission) (n = 22)	*P* value^a^	Percentage of patients with elevated levels during remission
Total Apo-A1	113.99 ± 16.35	169.64 ± 25.11	<0.001	95%
Total Apo-A2	20.63 ± 4.60	34.48 ± 4.85	<0.001	95%
Total Apo-B100	64.12 ± 13.64	86.92 ± 21.08	<0.001	82%
Total-PN	1,165.81 ± 248.08	1,580.49 ± 383.31	<0.001	82%
HDL-Apo-A2	22.00 ± 4.27	34.42 ± 4.60	<0.001	95%
HDL-TG	6.14 ± 1.55	10.85 ± 4.66	<0.001	95%
HDL-2-TG	0.93 ± 0.32	2.11 ± 0.97	<0.001	91%
HDL-3-TG	1.15 ± 0.36	1.91 ± 0.78	<0.001	77%
HDL-2-Apo-A2	1.80 ± 0.72	4.12 ± 1.12	<0.001	100%
HDL-3-Apo-A3	3.64 ± 1.41	7.03 ± 1.87	<0.001	95%
HDL-4-Apo-A2	13.74 ± 3.20	17.23 ± 5.58	<0.001	59%
IDL-CH	4.17 ± 2.39	11.23 ± 4.17	<0.001	86%
IDL-FC	1.21 ± 0.70	3.28 ± 1.17	<0.001	86%
IDL-PL	1.17 ± 1.61	7.15 ± 1.44	<0.001	100%
IDL-PN	34.81 ± 20.37	70.91 ± 28.24	<0.001	77%
IDL-Apo-B100	1.91 ± 1.12	3.9 ± 1.55	<0.001	77%
LDL-5-TG	1.02 ± 0.89	1.76 ± 0.95	0.010	73%
LDL-6-Apo-B100	13.30 ± 3.88	17.05 ± 5.87	0.016	73%
LDL-6-CH	15.72 ± 4.82	21.52 ± 7.46	0.004	82%
LDL-6-FC	3.94 ± 1.16	5.88 ± 1.82	<0.001	86%
LDL-6-PL	9.28 ± 2.50	12.42 ± 3.70	0.002	86%
LDL-6-PN	241.75 ± 70.60	310.05 ± 106.80	0.016	73%
VLDL-TG	34.41 ± 22.84	59.16 ± 33.59	0.007	77%
VLDL-1-FC	0.80 ± 0.76	1.81 ± 1.43	0.006	64%
VLDL-2-CH	0.99 ± 0.87	2.08 ± 1.26	0.002	73%
VLDL-2-FC	0.49 ± 0.42	1.16 ± 0.62	<0.001	73%
VLDL-2-PL	1.01 ± 0.94	2.51 ± 1.31	<0.001	86%
VLDL-2-TG	4.13 ± 3.91	9.57 ± 5.26	<0.001	82%
VLDL-3-CH	1.18 ± 0.74	3.06 ± 1.88	0.0001	77%
VLDL-3-FC	0.59 ± 0.41	1.42 ± 0.85	<0.001	77%
VLDL-3-PL	1.24 ± 0.87	3.37 ± 1.67	<0.001	82%
VLDL-3-TG	3.66 ± 2.88	9.98 ± 4.86	<0.001	82%
VLDL-4-CH	2.03 ± 1.27	3.77 ± 2.24	0.003	68%
VLDL-4-FC	0.76 ± 0.40	1.86 ± 0.95	<0.001	77%
VLDL-4-PL	2.00 ± 1.00	3.87 ± 1.66	<0.001	86%
VLDL-4-TG	4.37 ± 1.97	8.47 ± 3.34	<0.001	91%

a *P* value by Student’s *t-*test with false discovery rate correction.

## Discussion

**Fig. 5:**
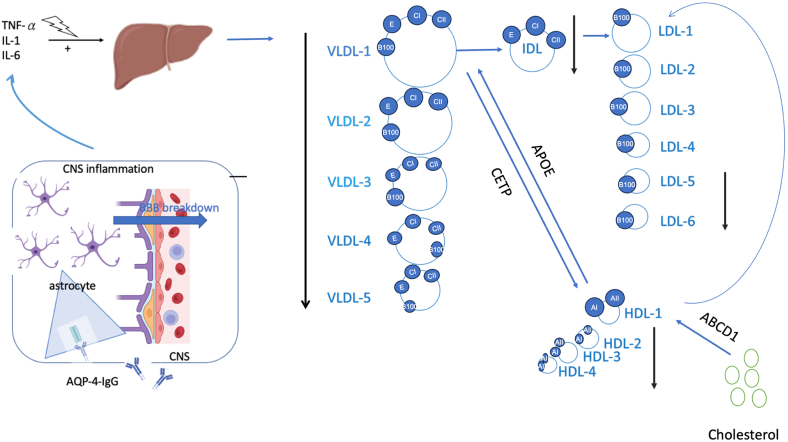
Proposed model of lipoprotein metabolism in NMOSD, illustrating the influence of proinflammatory cytokines and AQP4-IgG on increased VLDL production and impaired HDL-mediated cholesterol efflux during relapses. Proinflammatory cytokines (tumor necrosis factor-α, IL-1, and IL-6) trigger hepatic responses leading to VLDL production. CNS inflammation compromises the BBB, facilitated by astrocyte activation and autoantibodies targeting AQP-4-IgG. VLDL undergoes sequential conversion into IDL and LDL, contributing to cholesterol transport. HDL, regulated by APOE and cholesteryl ester transfer protein (CETP), facilitates cholesterol efflux, with ABCD1 (ATP-binding cassette subfamily D member 1) mediating cholesterol homeostasis. This pathway underscores the link between neuroinflammation, lipid metabolism, and cholesterol regulation in CNS disorders. Solid arrow icons: during relapsing.

This study presents the first comprehensive ^1^H-NMR-based lipidomic analysis of plasma lipoprotein profiles in NMOSD, identifying significant alterations in 39 of 112 lipoprotein components compared with MS patients and HCs. These components were significantly reduced during relapses in NMOSD, with 36 returning to levels comparable to HCs during remission. Notably, HDL-4-Apo-A2 levels showed a negative correlation with EDSS scores and the spinal cord lesion length. VLDL-3 and HDL-3 demonstrated the strongest discriminatory power in distinguishing NMOSD from both MS and HCs. These findings reveal a distinct dyslipidemia pattern in NMOSD and suggest the potential of lipoproteins as biomarkers for disease activity and differential diagnosis (Fig. 5).

HDLs are complex lipoproteins enriched in CH, PLs, Apo-A1, and Apo-A2, playing a pivotal role in lipid metabolism and cardiovascular diseases (24, 25). In the peripheral tissues, HDLs facilitate CH efflux to the liver for excretion (26). This process is primarily driven by Apo-A1, the major apolipoprotein in HDL, known for its protective effects against coronary heart disease (26, 27). Apo-A2, the second most abundant apolipoprotein, exerts both protective and detrimental effects. It potentially reduces atherosclerosis by enhancing hepatic lipase activity, yet can increase atherosclerotic risk by inhibiting lecithin-CH acyltransferase and interfering with hepatic CH uptake (28). Inflammation reduces HDL levels and alters lipoprotein size and composition (29). In NMOSD, reduced levels of multiple HDL components have been associated with increased disease activity and disability (30, 31, 32, 33, 34). An untargeted ^1^H-NMR-based lipoprotein study reported a reduction in small HDL particles and an increase in large HDL particles in NMOSD compared with MS (35). Furthermore, the monocyte-to-HDL ratio, a marker of inflammation and oxidative stress, correlates with NMOSD severity (34). Our findings further support this association, revealing significantly reduced Apo-A2 levels across multiple HDL subfractions in NMOSD patients. Importantly, HDL-4-Apo-A2 levels showed a negative correlation with EDSS scores and spinal cord lesion length, suggesting an association between HDL reduction and disease severity. The normalization of HDL components during remission implies that their reduction may result from inflammation-induced impairment of CH metabolism, warranting further investigation.

In plasma, TG-rich VLDLs are hydrolyzed by lipoprotein lipase to free fatty acids and glycerol, forming smaller VLDL remnants that become IDLs. IDLs are further hydrolyzed by hepatic lipase to form LDLs (36). Both acute and chronic inflammation disrupt this VLDL-IDL-LDL metabolic cascade. Acute infections significantly reduce both LDL and HDL CH levels (31), whereas chronic inflammatory diseases, such as rheumatoid arthritis, systemic lupus erythematosus, and psoriasis, are also associated with decreased LDL-C levels (37). Notably, immunological treatment of these inflammatory diseases restores lipid profiles, increasing both HDL and LDL levels (38). For example, short-term high-dose glucocorticoid treatment enhances hepatic VLDL secretion, leading to elevated serum TG and LDL levels (39). In our cohort, LDL levels, particularly small, dense LDL-5 and LDL-6 subfractions, were reduced during NMOSD relapses and returned to normal during remission. This normalization may reflect the resolution of inflammation. However, the potential effects of immunotherapies, such as prednisolone or azathioprine, on lipoprotein profiles were not fully assessed. Future studies will be needed to investigate the impact of immunotherapies on lipoprotein dynamics during acute relapse.

In the adult brain, CH synthesis primarily occurs in astrocytes and neurons and is transported by HDL (40). Brain CH metabolism is distinct from that of peripheral tissues, largely due to the restricted nature of blood-brain barrier (BBB). However, increasing evidence indicates that the BBB is disrupted during the active disease phase of NMOSD (41), potentially allowing the exchange of lipoprotein subclasses across the compromised BBB during a relapse. Although NMOSD is considered a CNS-targeted autoimmune disorder, systemic immune activation and peripheral inflammatory signals play an important role in its pathogenesis (42). Elevated levels of circulating cytokines and complement activation have been observed in patients with NMOSD (43). Serum levels of interleukin-10 (IL-10) and transforming growth factor-β1 are markedly elevated in new-onset NMOSD when compared with other neurological disorders (44). IL-6 is significantly elevated in both the serum and the cerebrospinal fluid of patients with NMOSD (45). Importantly, IL-6 is also known to reduce the integrity and functionality of the BBB (45). Therefore, the altered plasma lipoprotein profiles observed in our study are more likely a reflection of systemic immune dysregulation rather than a consequence of CNS inflammation alone.

In contrast to other inflammatory diseases where proinflammatory cytokines often increase VLDL levels (46), NMOSD patients showed reduced VLDL levels during relapses. TC and TG levels were also significantly lower in NMOSD patients compared with MS and HCs, suggesting that the decrease in VLDL may be associated with reduced TC. These observations require further validation.

Distinguishing NMOSD from MS is critical due to their overlapping clinical features but distinct treatment approaches. Previous studies have reported that worsening disability in MS is associated with higher baseline levels of LDL, TC, and TGs, whereas elevated HDL levels correlate with reduced acute inflammatory activity on MRI (14). Another study demonstrated a significant correlation between the number of enhancing brain lesions and elevated plasma levels of both TC and LDL-CH (33). However, these studies did not provide detailed insights into lipoprotein composition or its dynamic relationship with disease activity. In contrast, our study emphasizes the distribution and composition of lipoprotein subclasses, which reflect on not only lipid abundance but also the underlying metabolic and inflammatory states that influence their particle size, density, and composition. Specific subfractions, particularly VLDL-3, HDL-3, HDL-4-Apo-A2, may serve as a noninvasive tool for differential diagnosis and monitoring therapeutic responses. Given that this subclass analysis is clinically feasible, noninvasive, and easily standardized across laboratories, it represents a promising tool for routine clinical practice. Integrating lipoprotein profiling with cytokine panels or imaging biomarkers may further improve diagnostic accuracy. Future studies should explore the mechanistic links among AQP4-IgG, neuroinflammation, and lipid metabolism, as well as assess the impact of specific immunotherapies on lipoprotein profiles.

This study has several limitations. The relatively small sample size may have limited the ability to detect subtle changes in lipoprotein profiles. The potential impact of medications, especially glucocorticoids, on lipoprotein metabolism was not fully assessed, and the inclusion of nonfasting samples could have introduced variability. Large-scale longitudinal studies are needed to validate these findings and establish clinical thresholds for lipoprotein biomarkers in NMOSD.

## Data availability

The authors confirm that all the data supporting the findings of this study are included within the article. Raw data will be shared by the corresponding author on request.

## Supplemental data

This article contains supplemental data.

## Conflict of interest

The authors declare that they have no conflicts of interest with the contents of this article.

## References

[1] Kawachi I., Lassmann H. (2017). Neurodegeneration in multiple sclerosis and neuromyelitis optica. J. Neurol. Neurosurg. Psychiatry.

[2] Lennon V.A., Wingerchuk D.M., Kryzer T.J., Pittock S.J., Lucchinetti C.F., Fujihara K. (2004). A serum autoantibody marker of neuromyelitis optica: distinction from multiple sclerosis. Lancet.

[3] Lennon V.A., Kryzer T.J., Pittock S.J., Verkman A.S., Hinson S.R. (2005). IgG marker of optic-spinal multiple sclerosis binds to the aquaporin-4 water channel. J. Exp. Med..

[4] Weinshenker B.G. (2007). Neuromyelitis optica is distinct from multiple sclerosis. Arch. Neurol..

[5] Bukhari W., Barnett M.H., Prain K., Broadley S.A. (2012). Molecular pathogenesis of neuromyelitis optica. Int. J. Mol. Sci..

[6] Smith L.C., Pownall H.J., Gotto A.M. (1978). The plasma lipoproteins: structure and metabolism. Annu. Rev. Biochem..

[7] Feingold K.R. (2022). Lipid and lipoprotein metabolism. Endocrinol. Metab. Clin. North Am..

[8] Lappegard K.T., Kjellmo C.A., Hovland A. (2021). High-density lipoprotein subfractions: much Ado about nothing or clinically important?. Biomedicines.

[9] Packard C.J., Gaw A., Demant T., Shepherd J. (1995). Development and application of a multicompartmental model to study very low density lipoprotein subfraction metabolism. J. Lipid Res..

[10] Berneis K.K., Krauss R.M. (2002). Metabolic origins and clinical significance of LDL heterogeneity. J. Lipid Res..

[11] Ference B.A., Ginsberg H.N., Graham I., Ray K.K., Packard C.J., Bruckert E. (2017). Low-density lipoproteins cause atherosclerotic cardiovascular disease. 1. Evidence from genetic, epidemiologic, and clinical studies. A consensus statement from the European Atherosclerosis Society Consensus Panel. Eur. Heart J..

[12] Camont L., Chapman M.J., Kontush A. (2011). Biological activities of HDL subpopulations and their relevance to cardiovascular disease. Trends Mol. Med..

[13] Giubilei F., Antonini G., Di Legge S., Sormani M.P., Pantano P., Antonini R. (2002). Blood cholesterol and MRI activity in first clinical episode suggestive of multiple sclerosis. Acta Neurol. Scand..

[14] Weinstock-Guttman B., Zivadinov R., Mahfooz N C.E., Drake A., Schneider J., Teter B. (2011). Serum lipid profiles are associated with disability and MRI outcomes in multiple sclerosis. J. Neuroinflammation.

[15] Browne R.W., Weinstock-Guttman B., Horakova D., Zivadinov R., Bodziak M.L., Tamano-Blanco M. (2014). Apolipoproteins are associated with new MRI lesions and deep grey matter atrophy in clinically isolated syndromes. J. Neurol. Neurosurg. Psychiatry.

[16] Tettey P., Simpson S., Taylor B., Blizzard L., Ponsonby A.L., Dwyer T. (2014). An adverse lipid profile is associated with disability and progression in disability, in people with MS. Mult. Scler..

[17] Murali N., Browne R.W., Fellows Maxwell K., Bodziak M.L., Jakimovski D., Hagemeier J. (2020). Cholesterol and neurodegeneration: longitudinal changes in serum cholesterol biomarkers are associated with new lesions and gray matter atrophy in multiple sclerosis over 5 years of follow-up. Eur. J. Neurol..

[18] Newcombe J., Li H., Cuzner M.L. (1994). Low density lipoprotein uptake by macrophages in multiple sclerosis plaques- implications for pathogenesis. Neuropathol. Appl. Neurobiol..

[19] Wingerchuk D.M., Banwell B., Bennett J.L., Cabre P., Carroll W., Chitnis T. (2015). International consensus diagnostic criteria for neuromyelitis optica spectrum disorders. Neurology.

[20] Thompson A.J., Banwell B.L., Barkhof F., Carroll W.M., Coetzee T., Comi G. (2018). Diagnosis of multiple sclerosis: 2017 revisions of the McDonald criteria. Lancet Neurol..

[21] Kurtzke J.F. (1983). Rating neurologic impairment in multiple sclerosis: an expanded disability status scale (EDSS). Neurology.

[22] Jiménez B., Holmes E., Heude C., Tolson R.F., Harvey N., Lodge S.L. (2018). Quantitative lipoprotein subclass and low molecular weight metabolite analysis in human serum and plasma by 1H NMR spectroscopy in a multilaboratory trial. Anal. Chem..

[23] Loo R.L., Lodge S., Kimhofer T., Bong S.H., Begum S., Whiley L. (2020). Quantitative in-vitro diagnostic NMR spectroscopy for lipoprotein and metabolite measurements in plasma and serum: recommendations for analytical artifact minimization with special reference to COVID-19/SARS-CoV-2 samples. J. Proteome Res..

[24] Thakkar H., Vincent V., Sen A., Singh A., Roy A. (2021). Changing perspectives on HDL: from simple quantity measurements to functional quality assessment. J. Lipids.

[25] Chang K.H., Cheng M.L., Lo C.J., Fan C.M., Wu Y.R., Chen C.M. (2023). Alternations of lipoprotein profiles in the plasma as biomarkers of huntington's disease. Cells.

[26] Oram J.F., Yokoyama S. (1996). Apolipoprotein-mediated removal of cellular cholesterol and phospholipids. J. Lipid Res..

[27] Birjmohun R.S., Dallinga-Thie G.M., Kuivenhoven J.A., Stroes E.S., Otvos J.D., Wareham N.J. (2007). Apolipoprotein A-II is inversely associated with risk of future coronary artery disease. Circulation.

[28] Florea G., Tudorache I.F., Fuior E.V., Ionita R., Dumitrescu M., Fenyo I.M. (2022). Apolipoprotein A-II, a player in multiple processes and diseases. Biomedicines.

[29] Esteve E., Ricart W., Fernandez-Real J.M. (2005). Dyslipidemia and inflammation: an evolutionary conserved mechanism. Clin. Nutr..

[30] Li Y., Wang H., Hu X., Peng F., Yang Y. (2010). Serum lipoprotein levels in patients with neuromyelitis optica elevated but had little correlation with clinical presentations. Clin. Neurol. Neurosurg..

[31] Zhong Y.H., Liu J., Li M., Wang X., Yuan Y., Zhong X.F. (2013). Distinct serum apolipoprotein AI levels in neuromyelitis optica and acute transverse myelitis. Lipids Health Dis..

[32] Cho E.B., Cho H.J., Choi M., Seok J.M., Shin H.Y., Kim B.J. (2020). Low high-density lipoprotein cholesterol and high triglycerides lipid profile in neuromyelitis optica spectrum disorder: associations with disease activity and disability. Mult. Scler. Relat. Disord..

[33] Thoman M.E., McKarns S.C. (2020). Metabolomic profiling in neuromyelitis optica spectrum disorder biomarker discovery. Metabolites.

[34] Zhang J., Li Y., Zhou Y., Wang K., Pan C., Zhao Y. (2021). Monocyte to high-density lipoprotein ratio: a novel predictive marker of disease severity and prognosis in patients with neuromyelitis optica spectrum disorders. Front. Neurol..

[35] Jurynczyk M., Probert F., Yeo T., Tackley G., Claridge T.D.W., Cavey A. (2017). Metabolomics reveals distinct, antibody-independent, molecular signatures of MS, AQP4-antibody and MOG-antibody disease. Acta Neuropathol. Commun..

[36] Kwiterovich P.O.J. (2000). The metabolic pathways of high-density lipoprotein, low-density lipoprotein, and triglycerides- A current review. Am. J. Cardiol..

[37] Feingold K.R., Grunfeld C., Feingold Endotext.K.R., Ahmed S.F., Anawalt B., Blackman M.R., Boyce A., Chrousos G. (2000). MDText.com, Inc. Copyright © 2000-2025.

[38] Park Y.B., Choi H.K., Kim M.Y., Lee W.K., Song J., Kim D.K. (2002). Effects of antirheumatic therapy on serum lipid levels in patients with rheumatoid arthritis: a prospective study. Am. J. Med..

[39] Ettinger W.H., Hazzard W.R. (1988). Prednisone increases very low density lipoprotein and high density lipoprotein in healthy men. Metabolism.

[40] Vance J.E., Hayashi H., Karten B. (2005). Cholesterol homeostasis in neurons and glial cells. Semin. Cell Dev. Biol..

[41] Carnero Contentti E., Correale J. (2021). Neuromyelitis optica spectrum disorders: from pathophysiology to therapeutic strategies. J. Neuroinflammation.

[42] Xie Z., Zhou Q., Hu J., He L., Meng H., Liu X. (2024). Integrated omics profiling reveals systemic dysregulation and potential biomarkers in the blood of patients with neuromyelitis optica spectrum disorders. J. Transl. Med..

[43] Bauer A., Rudzki D., Berek K., Dinoto A., Lechner C., Wendel E.M. (2022). Increased peripheral inflammatory responses in myelin oligodendrocyte glycoprotein associated disease and aquaporin-4 antibody positive neuromyelitis optica spectrum disorder. Front. Immunol..

[44] Wei Y., Chang H., Li X., Wang H., Du L., Zhou H. (2018). Cytokines and tissue damage biomarkers in first-onset neuromyelitis optica spectrum disorders: significance of interleukin-6. Neuroimmunomodulation.

[45] Fujihara K., Bennett J.L., de Seze J., Haramura M., Kleiter I., Weinshenker B.G. (2020). Interleukin-6 in neuromyelitis optica spectrum disorder pathophysiology. Neurol. Neuroimmunol. Neuroinflamm..

[46] Khovidhunkit W., Kim M.S., Memon R.A., Shigenaga J.K., Moser A.H., Feingold K.R. (2004). Effects of infection and inflammation on lipid and lipoprotein metabolism: mechanisms and consequences to the host. J. Lipid Res..

