# Supplementary material for: Lipoprotein dynamics in neuromyelitis optica spectrum disorder
Source: J Lipid Res. 2025 Jul 21;66(8):100864. doi: 10.1016/j.jlr.2025.100864 (PMC12391798; doi:10.1016/j.jlr.2025.100864)
Supplement: Supplementary Material [file mmc1.docx]

**Supplementary Table 1. Comprehensive lipoprotein plasma levels for patients with neuromyelitis optica spectrum disorder (NMOSD) compared to multiple sclerosis (MS) and healthy controls (HCs).**

| Metabolite  (mg/dL, mean ± SD) | HC  (n=41) | NMOSD  (n=40) | MS  (n=35) | *P* value^*^  (3 groups) | *P* value^**^ (NMOSD vs. HC) | *P* value^***^  (NMOSD vs. MS) |
| --- | --- | --- | --- | --- | --- | --- |
| ABA1 | 0.56 ± 0.17 | 0.57 ± 0.15 | 0.58 ± 0.16 | 0.877 | 0.8 | 0.785 |
| HDL-CH | 53.33 ± 13.96 | 48.36 ± 12.50 | 51.78 ± 12.27 | 0.217 | 0.096 | 0.237 |
| HDL-FC | 12.00 ± 3.35 | 12.32 ± 3.20 | 12.51 ± 3.27 | 0.784 | 0.655 | 0.801 |
| HDL-PL | 71.02 ± 16.83 | 66.75 ± 13.61 | 69.74 ± 17.33 | 0.470 | 0.213 | 0.404 |
| HDL-Apo-A1 | 135.21 ± 28.32 | 118.96 ± 23.45 | 129.57 ± 21.92 | 0.014 | 0.006 | 0.05 |
| HDL-1-TG | 3.72 ± 2.28 | 3.19 ± 2.01 | 4.3 8± 2.14 | 0.062 | 0.272 | 0.016 |
| HDL-1-CH | 17.46 ± 6.96 | 18.08 ± 7.92 | 20.02 ± 9.31 | 0.365 | 0.705 | 0.334 |
| HDL-1-FC | 3.58 ± 1.70 | 3.59 ± 1.61 | 4.03 ± 1.88 | 0.438 | 0.967 | 0.276 |
| HDL-1-PL | 20.12 ± 8.69 | 21.20 ± 9.89 | 23.79 ± 11.62 | 0.274 | 0.601 | 0.300 |
| HDL-1-Apo-A1 | 24.20 ± 12.55 | 26.37 ± 13.90 | 30.67 ± 16.07 | 0.139 | 0.463 | 0.219 |
| HDL-1-Apo-A2 | 2.16 ± 1.40 | 2.11 ± 1.12 | 2.70 ± 1.65 | 0.142 | 0.872 | 0.074 |
| HDL-2-Apo-A1 | 16.91 ± 4.38 | 15.39 ± 3.58 | 17.11 ± 5.12 | 0.171 | 0.092 | 0.094 |
| HDL-2-PL | 12.63 ± 3.91 | 11.42 ± 3.13 | 12.58 ± 2.65 | 0.231 | 0.132 | 0.123 |
| HDL-2-CH | 8.19 ± 2.75 | 6.97 ± 2.46 | 8.01 ± 2.54 | 0.079 | 0.038 | 0.075 |
| HDL-2-FC | 1.49 ± 0.77 | 1.30 ± 0.58 | 1.40 ± 0.57 | 0.418 | 0.21 | 0.423 |
| HDL-3-CH | 9.30 ± 2.82 | 7.40 ± 2.10 | 8.21 ± 1.86 | 0.002 | 0.001 | 0.085 |
| HDL-3-FC | 1.69 ± 0.77 | 1.23 ± 0.57 | 1.34 ± 0.57 | 0.006 | 0.003 | 0.416 |
| HDL-3-PL | 14.68 ± 3.94 | 12.58 ± 2.65 | 13.41 ± 3.19 | 0.019 | 0.006 | 0.22 |
| HDL-3-Apo-A1 | 25.43 ± 6.16 | 21.70 ± 4.36 | 23.73 ± 5.30 | 0.009 | 0.002 | 0.073 |
| HDL-4-CH | 17.75 ± 5.67 | 14.90 ± 4.03 | 14.18 ± 3.77 | 0.002 | 0.011 | 0.428 |
| HDL-4-FC | 3.28 ± 1.30 | 2.54 ± 1.00 | 2.37 ± 1.07 | 0.001 | 0.006 | 0.474 |
| HDL-4-PL | 23.52 ± 7.08 | 21.25 ± 4.74 | 19.98 ± 4.73 | 0.025 | 0.095 | 0.25 |
| HDL-4-TG | 3.36 ± 1.01 | 2.72 ± 1.00 | 3.16 ± 1.08 | 0.020 | 0.005 | 0.074 |
| HDL-4-Apo-A1 | 67.46 ± 17.94 | 57.31 ± 11.79 | 57.42 ± 10.70 | 0.002 | 0.004 | 0.965 |
| LDHD | 1.66 ± 0.55 | 1.52 ± 0.53 | 1.53 ± 0.54 | 0.431 | 0.241 | 0.922 |
| IDL-TG | 9.86 ± 15.54 | 5.91 ± 4.64 | 63.58 ± 11.97 | 0.052 | 0.127 | 0.001 |
| LDL-CH | 86.66 ± 29.84 | 71.73 ± 23.96 | 75.90 ± 23.03 | 0.032 | 0.015 | 0.447 |
| LDL-PN | 1075.43 ± 297.62 | 970.62 ± 239.43 | 1027.83 ± 252.84 | 0.210 | 0.085 | 0.318 |
| LDL-TG | 17.26 ± 5.92 | 16.25 ± 5.46 | 11.97 ± 9.20 | 0.151 | 0.427 | 0.051 |
| LDL-FC | 25.72 ± 8.03 | 23.91 ± 6.59 | 24.05 ± 6.30 | 0.444 | 0.271 | 0.928 |
| LDL-PL | 50.89 ± 13.87 | 44.98 ± 11.34 | 45.75 ± 12.61 | 0.080 | 0.039 | 0.782 |
| LDL-Apo-B100 | 59.15 ± 16.37 | 53.38 ± 13.17 | 56.53 ± 13.90 | 0.210 | 0.085 | 0.318 |
| LDL-1-PN | 204.79 ± 66.02 | 221.10 ± 65.03 | 220.86 ± 75.98 | 0.482 | 0.266 | 0.988 |
| LDL-1-TG | 5.94 ± 2.70 | 6.66 ± 2.55 | 7.24 ± 2.79 | 0.110 | 0.221 | 0.352 |
| LDL-1-CH | 20.29 ± 6.95 | 20.48 ± 7.08 | 21.18 ± 7.05 | 0.849 | 0.9 | 0.672 |
| LDL-1-FC | 6.01 ± 2.07 | 6.02 ± 2.14 | 6.32 ± 2.06 | 0.766 | 0.986 | 0.533 |
| LDL-1-PL | 11.89 ± 3.61 | 12.28 ± 3.60 | 12.26 ± 4.22 | 0.874 | 0.626 | 0.982 |
| LDL-1-Apo-B100 | 11.26 ± 3.63 | 12.16 ± 3.58 | 12.15 ± 4.18 | 0.483 | 0.266 | 0.988 |
| LDL-2-Apo-B100 | 7.94 ± 3.15 | 10.52 ± 3.20 | 9.75 ± 3.40 | 0.002 | <0.001 | 0.317 |
| LDL-2-PL | 8.02 ± 3.23 | 10.25 ± 3.23 | 9.43 ± 3.62 | 0.012 | 0.003 | 0.302 |
| LDL-2-FC | 4.58 ± 1.99 | 5.57 ± 2.02 | 5.28 ± 2.08 | 0.083 | 0.029 | 0.55 |
| LDL-2-CH | 13.95 ± 6.31 | 18.29 ± 6.59 | 16.89 ± 6.94 | 0.012 | 0.003 | 0.372 |
| LDL-2-PN | 144.38 ± 57.24 | 191.20 ± 58.12 | 177.68 ± 61.45 | 0.002 | <0.001 | 0.331 |
| LDL-2-TG | 2.23 ± 0.83 | 2.33 ± 0.83 | 2.41 ± 0.91 | 0.678 | 0.611 | 0.697 |
| LDL-3-PN | 134.01 ± 61.29 | 138.74 ± 47.36 | 127.84 ± 56.68 | 0.697 | 0.699 | 0.367 |
| LDL-3-TG | 2.14 ± 0.65 | 2.70 ± 0.73 | 2.47 ± 0.80 | 0.003 | < 0.001 | 0.192 |
| LDL-3-CH | 12.40 ± 6.19 | 11.86 ± 5.33 | 10.72 ± 5.95 | 0.446 | 0.671 | 0.384 |
| LDL-3-FC | 4.52 ± 2.06 | 4.55 ± 1.49 | 4.03 ± 1.84 | 0.394 | 0.943 | 0.184 |
| LDL-3-PL | 7.22 ± 3.19 | 7.16 ± 2.59 | 6.46 ± 3.01 | 0.475 | 0.921 | 0.287 |
| LDL-3-Apo-B100 | 7.37 ± 3.37 | 7.63 ± 2.60 | 7.01 ± 3.13 | 0.680 | 0.697 | 0.352 |
| LDL-4-Apo-B100 | 6.68 ± 3.41 | 3.85 ± 2.54 | 3.93 ± 2.88 | < 0.001 | <0.001 | 0.902 |
| LDL-4-PL | 6.40 ± 3.15 | 3.80 ± 2.31 | 3.68 ± 2.54 | < 0.001 | <0.001 | 0.832 |
| LDL-4-FC | 3.70 ± 1.80 | 2.38 ± 1.20 | 2.15 ± 1.34 | < 0.001 | <0.001 | 0.435 |
| LDL-4-CH | 11.12 ± 5.84 | 6.57 ± 3.70 | 6.04 ± 4.60 | < 0.001 | <0.001 | 0.581 |
| LDL-4-TG | 1.88 ± 0.87 | 1.39 ± 0.94 | 1.55 ± 0.83 | 0.044 | 0.018 | 0.457 |
| LDL-4-PN | 121.05 ± 62.37 | 70.28 ± 46.05 | 71.12 ± 52.13 | < 0.001 | <0.001 | 0.942 |
| LDL-5-PN | 150.79 ± 88.70 | 81.32 ± 60.97 | 107.19 ± 64.58 | < 0.001 | <0.001 | 0.076 |
| LDL-5-CH | 11.70 ± 7.23 | 5.77 ± 4.67 | 7.60 ± 4.94 | < 0.001 | <0.001 | 0.105 |
| LDL-5-FC | 3.43 ± 1.80 | 2.02 ± 1.24 | 2.41 ± 1.39 | < 0.001 | <0.001 | 0.211 |
| LDL-5-PL | 6.58 ± 3.59 | 3.71 ± 2.44 | 4.38 ± 2.64 | < 0.001 | <0.001 | 0.256 |
| LDL-5-Apo-B100 | 8.29 ± 4.88 | 4.47 ± 3.35 | 5.89 ± 3.50 | < 0.001 | <0.001 | 0.077 |
| LDL-6-TG | 3.44 ± 1.43 | 3.25 ± 1.13 | 3.83 ± 1.37 | 0.163 | 0.5 | 0.05 |
| VLDL-Apo-B100 | 8.31 ± 4.91 | 6.64 ± 3.02 | 9.50 ± 4.61 | 0.160 | 0.069 | 0.002 |
| VLDL-PN | 151.14±89.31 | 120.65 ± 54.99 | 172.67 ± 83.77 | 0.016 | 0.069 | 0.002 |
| VLDL-1-TG | 40.24 ± 55.99 | 24.79 ± 20.56 | 50.22 ± 43.26 | 0.038 | 0.105 | 0.001 |
| VLDL-1-CH | 7.91 ± 10.79 | 5.22 ± 3.50 | 10.19 ± 8.58 | 0.035 | 0.137 | 0.001 |
| VLDL-1-PL | 6.00 ± 7.92 | 3.49 ± 2.84 | 6.72 ± 5.43 | 0.042 | 0.063 | 0.002 |
| VLDL-5-TG | 3.21 ± 0.94 | 3.55 ± 0.93 | 3.60 ± 0.98 | 0.145 | 0.105 | 0.831 |
| VLDL-5-CH | 1.64 ± 0.69 | 2.06 ± 0.83 | 1.82 ± 0.77 | 0.057 | 0.018 | 0.208 |
| VLDL-5-FC | 0.95 ± 0.92 | 1.07 ± 0.47 | 1.35 ± 1.05 | 0.120 | 0.463 | 0.138 |
| VLDL-5-PL | 2.08 ± 0.72 | 2.34 ± 0.83 | 2.29 ± 0.72 | 0.271 | 0.135 | 0.77 |

ABA1, Apolipoprotein B100/Apolipoprotein A1 ratio;  Apo, apolipoprotein; CH, total cholesterol; free cholesterol; HDL, high-density lipoprotein; IDL, Intermediate-density lipoprotein; LDHD, low-density lipoprotein-cholesterol/high-density lipoprotein-cholesterol ratio; PL, phospholipid; TG triglyceride. VLDL, very-low-density lipoprotein;

^*^*P* value by ANOVA with FDR correction.

*^**^P value* by Bonferroni *post hoc* adjustment.

**^***^***P* value by Bonferroni *post hoc* adjustment.
